# Non-monophyly and intricate morphological evolution within the avian family Cettiidae revealed by multilocus analysis of a taxonomically densely sampled dataset

**DOI:** 10.1186/1471-2148-11-352

**Published:** 2011-12-05

**Authors:** Per Alström, Sebastian Höhna, Magnus Gelang, Per GP Ericson, Urban Olsson

**Affiliations:** 1 Swedish Species Information Centre, Swedish University of Agricultural Sciences, Box 7007, SE-750 07 Uppsala, Sweden; 2 Department of Mathematics, Stockholm University, SE-106 91 Stockholm, Sweden; 3 Department of Vertebrate Zoology, and Molecular Systematics Laboratory, Swedish Museum of Natural History, P.O. Box 50007, SE-104 05 Stockholm, Sweden; 4 Department of Zoology, University of Gothenburg, Box 463, SE-405 30 Göteborg, Sweden

## Abstract

**Background:**

The avian family Cettiidae, including the genera *Cettia*, *Urosphena*, *Tesia*, *Abroscopus *and *Tickellia *and *Orthotomus cucullatus*, has recently been proposed based on analysis of a small number of loci and species. The close relationship of most of these taxa was unexpected, and called for a comprehensive study based on multiple loci and dense taxon sampling. In the present study, we infer the relationships of all except one of the species in this family using one mitochondrial and three nuclear loci. We use traditional gene tree methods (Bayesian inference, maximum likelihood bootstrapping, parsimony bootstrapping), as well as a recently developed Bayesian species tree approach (*BEAST) that accounts for lineage sorting processes that might produce discordance between gene trees. We also analyse mitochondrial DNA for a larger sample, comprising multiple individuals and a large number of subspecies of polytypic species.

**Results:**

There are many topological incongruences among the single-locus trees, although none of these is strongly supported. The multi-locus tree inferred using concatenated sequences and the species tree agree well with each other, and are overall well resolved and well supported by the data. The main discrepancy between these trees concerns the most basal split. Both methods infer the genus *Cettia *to be highly non-monophyletic, as it is scattered across the entire family tree. Deep intraspecific divergences are revealed, and one or two species and one subspecies are inferred to be non-monophyletic (differences between methods).

**Conclusions:**

The molecular phylogeny presented here is strongly inconsistent with the traditional, morphology-based classification. The remarkably high degree of non-monophyly in the genus *Cettia *is likely to be one of the most extraordinary examples of misconceived relationships in an avian genus. The phylogeny suggests instances of parallel evolution, as well as highly unequal rates of morphological divergence in different lineages. This complex morphological evolution apparently misled earlier taxonomists. These results underscore the well-known but still often neglected problem of basing classifications on overall morphological similarity. Based on the molecular data, a revised taxonomy is proposed. Although the traditional and species tree methods inferred much the same tree in the present study, the assumption by species tree methods that all species are monophyletic is a limitation in these methods, as some currently recognized species might have more complex histories.

## Background

In a study of large-scale relationships within the avian superfamily Sylvioidea, Alström *et al*. [1] found, based on mitochondrial cytochrome *b *and nuclear myoglobin intron 2 sequence data, that two species of *Cettia *and one species each of *Urosphena*, *Tesia*, *Abroscopus *and *Tickellia*, and *Orthotomus cucullatus *formed a clade, well separated from a broad selection of other passerines. They proposed the family name Cettiidae for this group. This clade (limited to one species each of *Cettia*, *Abroscopus *and *Tickellia*) was corroborated by Johansson *et al*. [2] based on myoglobin, ornithine decarboxylase (ODC), and ß-fibrinogen introns. Irestedt *et al*. [3] concluded, based on all of the previously used loci, but with a glyceraldehyde-3-phosphodehydrogenase (GAPDH) intron instead of ß-fibrinogen, that *Hemitesia *was also part of this clade. Two of the above studies [1,3] indicated that the genus *Cettia *is non-monophyletic. Most of these findings were entirely unexpected based on the traditional, morphology-based classification, although *Cettia *and *Urosphena *have long been considered closely related, and some species have been moved back and forth between these genera (cf. e.g. [4-12]).

Altogether, nearly 95 taxa are recognised in Cettiidae, separated into 25-29 species [7,9,12,13]. Two of the species have been described in the last 25 years, namely *Cettia carolinae *Rozendaal, 1987 [14] and *Cettia haddeni *LeCroy and Barker, 2006 [15]. The genus *Cettia *has often been divided into subgenera, although there has been poor agreement between authors regarding the inclusiveness of these subgenera (e.g. [4,7]). As has already been indicated above, the generic allocation of some taxa has varied over time. At the species level, the taxonomy of several taxa has been disputed. *Cettia diphone *has variously been treated as a single species, or split into *C. diphone sensu stricto *and *C. canturians*, generally without providing any justification for either standpoint (cf. [4-7,9-13,16]). Furthermore, *Cettia seebohmi *has often been treated as a subspecies of *C. diphone sensu lato *(e.g. [4,7,11]), although some authors considered *C. seebohmi *to be a separate species, based on unpublished differences in song and lack of the pronounced sexual size dimorphism of *C. diphone*/*C. canturians *[5,10,12]. The latter position was later supported based on vocalizations and mitochondrial DNA [17]. Alström *et al*. [18] suggested, based on a study of morphology, vocalizations and mitochondrial DNA, that *Cettia acanthizoides *was better treated as two species, *C. acanthizoides sensu stricto *and *C. brunnescens*. Olsson *et al*. [19] proposed, based on analyses of mitochondrial and nuclear DNA, that some of the subspecies of *Cettia flavolivacea *be moved to *C. vulcania*. Bairlein *et al*. [13] treated *Orthotomus cucullatus heterolaemus *as a distinct species.

The species in the genera *Cettia *and *Urosphena *are nondescript, brown above and paler below, usually with a brownish, greyish or yellowish wash to the underparts, and have medium-length (*Cettia*) or short (*Urosphena*) tails (e.g. [13,16]). The various species of *Cettia *are generally more easily separable by voice than by external features [13,16]. *Oligura*, *Hemitesia *and *Tesia *are extremely short-tailed, and the two former and one of the *Tesia *species are comparatively colourful, with green and yellow colours [13,16]. *Abroscopus*, *Tickellia *and *Orthotomus cucullatus *are even more brightly coloured, with green, yellow and often bright rufous hues, and have medium-length tails [13]. All species in Cettiidae are sexually monomorphic, although some *Cettia *show pronounced sexual size dimorphism, and in most species juveniles resemble adults [13,16]. All Cettiidae have 10 rectrices (or eight, in the extremely short-tailed *Tesia*), unlike nearly all other passerines, which have 12 [1,3]. Illustrations of representatives of the different "morphotypes" are shown in the last figure in the paper.

Most species in Cettiidae occur in southern and eastern Asia, but *Hemitesia *is restricted to the Albertine Rift in East Africa, one *Cettia *extends its range to Europe and North Africa, and several species occur on Pacific islands. The majority are either sedentary or altitudinal migrants, but the most northerly breeding species are medium-distance migrants. Most species inhabit bushy areas, bamboo or forest undergrowth in mountains and foothills, although a few *Cettia *breed to above the tree limit or close to sea-level. All are insectivorous. [13,16]

The results of some recent studies [1-3] emphasize the need for a comprehensive analysis of Cettiidae based on a denser taxon sampling and multiple loci. In the present study, we infer the relationships of all except one of the species in the family using one mitochondrial gene and three nuclear introns (only mitochondrial data for three species). We use traditional gene tree methods (Bayesian inference, maximum likelihood bootstrapping, parsimony bootstrapping), as well as a recently developed Bayesian species tree approach (*BEAST; [20]) that accounts for lineage sorting processes that might produce discordance between gene trees. We also analyse mitochondrial DNA for a larger sample, comprising multiple individuals and a large number of subspecies of polytypic species. A revised classification is proposed based on our results.

## Results

### Sequence characteristics

We obtained a contiguous ≤724 base pair (bp) stretch of ODC, ≤707 bp of myo, ≤378 bp of GAPDH and ≤1078 bp of cyt*b*. No unexpected stop codons, indels or distinct double peaks in the chromatograms that would indicate the presence of nuclear pseudogenes were found in the coding cyt*b *sequences. The aligned ODC sequences comprised 732 characters, of which 173 (24%) were parsimony-informative; myo 727 characters, 120 (16.5%) parsimony-informative; GAPDH 386 characters, 86 (22%) parsimony-informative; and cyt*b *including all sequences 1078 characters, 391 (36%) parsimony-informative. The complete dataset contained 2923 characters, of which 769 (26%) were parsimony-informative.

### Model selection

In the analysis using MrBayes we selected models *a priori*. For the *BEAST analysis we used the same selected models and additionally a variety of models which are *BEAST-specific, such as the relaxed clock model. To establish how well each model fit the data, we calculated Bayes Factors (BF; [21,22]) using the harmonic mean as an approximation of the marginal likelihood of a model. The results from the BF analyses are given in Table 1 and Table 2. According to these comparisons, the partitioned MrBayes analysis of the concatenated data has a significantly higher marginal likelihood than the unpartitioned analysis of the same data. In all pairwise comparisons of the *BEAST analyses, the relaxed clock models scored higher than the strict clock models (all else being equal), showing evidence under all substitution models of violation of a strict molecular clock. The *BEAST analysis with the highest likelihood according to the BF comparison was the model in which all subspecies of a species were predefined as belonging to the same species, all loci had independent substitution models, and a relaxed clock prior was applied ("jModelTest relaxed"). Of the *BEAST analyses in which the individuals classified as the same subspecies were grouped *a priori *but were not predefined as belonging to the same species, the analysis with the locus-specific models and a relaxed clock prior ("Subspecies jModelTest relaxed") had the highest BF, although it was not strongly different from the analysis with a strict clock.

**Table 1:** log_10 _Bayes Factors for MrBayes analyses

Model	ln P(model | data)	Unpartitioned	Partitioned
Unpartitioned	-19982, 729	-	-212, 878
Partitioned	-19492, 56	212, 878	-

Calculated for partitioned and unpartitioned MrBayes analyses, respectively.

**Table 2:** log_10 _Bayes Factors for *BEAST analyses

Model	log_10 _P (model | data)	GTR relaxed	GTR strict	jModel Test relaxed	jModel Test strict	Subspecies GTR relaxed	Subspecies GTR strict	Subspecies jModelTest relaxed	Subspecies jModelTest strict
GTR relaxed	-6933.66	-	15.86	-24.50	-10.45	4.39	18.33	-20.90	-6.59
GTR strict	-6949.51	-15.86	-	-40.36	-26.30	-11.47	2.47	-36.75	-22.44
**jModelTest relaxed**	**-6909.16**	**24.50**	**40.36**	-	**14.05**	**28.89**	**42.83**	**3.61**	**17.92**
jModelTest strict	-6923.21	10.45	26.30	-14.05	-	14.83	28.77	-10.45	3.86
Subspecies GTR relaxed	-6938.04	-4.39	11.47	-28.89	-14.83	-	13.94	-25.28	-10.97
Subspecies GTR strict	-6951.98	-18.33	-2.47	-42.83	-28.77	-13.94	-	-39.22	-24.91
Subspecies jModelTest relaxed	-6912.76	20.90	36.75	-3.61	10.45	25.28	39.22	-	14.31
Subspecies jModelTest strict	-6927.07	6.59	22.44	-17.92	-3.86	10.97	24.91	-14.31	-

Calculated for *BEAST analyses under different models. "GTR" refers to analyses where cyt*b *was analysed under the GTR+Γ+I model and the other sequences under the HKY model; "jModelTest" refers to analyses under the models selected by jModelTest; "strict" refers to a strict molecular clock; and "relaxed" refers to an uncorrelated lognormal distributed relaxed clock. All analyses included all available sequences, i.e. also individuals for which only cyt*b *was available.

### MrBayes analyses

The single-locus analyses resulted in variously well resolved and well supported trees (cyt*b *most resolved, GAPDH least resolved; Additional files 1, 2, 3 and 4). Although there is much incongruence among the trees, no conflicting nodes are strongly supported.

**Figure 1:**
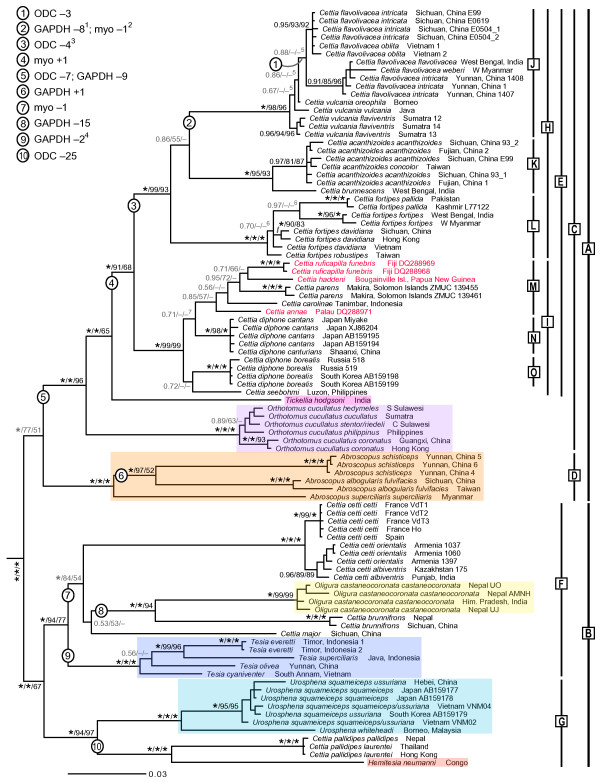
**Tree of Cettiidae based on concatenated sequences - all taxa, partitioned analysis**. Majority rule (50%) consensus tree, based on concatenated nuclear ODC, myoglobin and GAPDH and mitochondrial cytochrome *b*, inferred by Bayesian inference (BI), analysed in four partitions. All available sequences (including all subspecies) were included. Generic affinity according to traditional taxonomy [12] is indicated by different colour shadings. Labelled bars denote clades discussed in text. The three species for which only cytochrome *b *is available are in red. Posterior probabilities, and maximum likelihood (ML) and parsimony (MP) bootstrap values are indicated at the nodes, in this order; an asterisk represents posterior probability (PP) 1.00 or bootstrap 100%, and nodes with PP < 0.95 and/or conflicts between BI, ML and MP are in grey. The outgroups (*Alauda arvensis *and *Mirafra javanica *in Alaudidae and *Orthotomus sepium*, *O. sutorius *and *Prinia familiaris *in Cisticolidae) have been pruned from the tree. Numbers on internal branches refer to indels. ^1^Also in *Cettia cetti *and *C. fortipes fortipes *W Myanmar. ^2^Same position as number 4. ^3^Also in *Tickellia hodgsoni*. ^4^Also in *Cettia major*. ^5^According to MP, *C. vulcania vulcania *and *C. vulcania oreophila *form a trichotomy with *C. flavolivacea intricata *Sichuan/*C. flavolivacea oblita *(54%), and these are sisters to *C. vulcania flaviventris *(98%). ^6^According to MP, *C. fortipes pallida *is sister to the other *C. fortipes *subspecies (74%). According to ML, *C. fortipes davidiana *Vietnam and *C. fortipes robustipes *are sisters (62%), and these are sisters to *C. fortipes davidiana *Sichuan/Hong Kong (65%); according to MP, these relationships receive 71% and 100% support, respectively.

**Figure 2:**
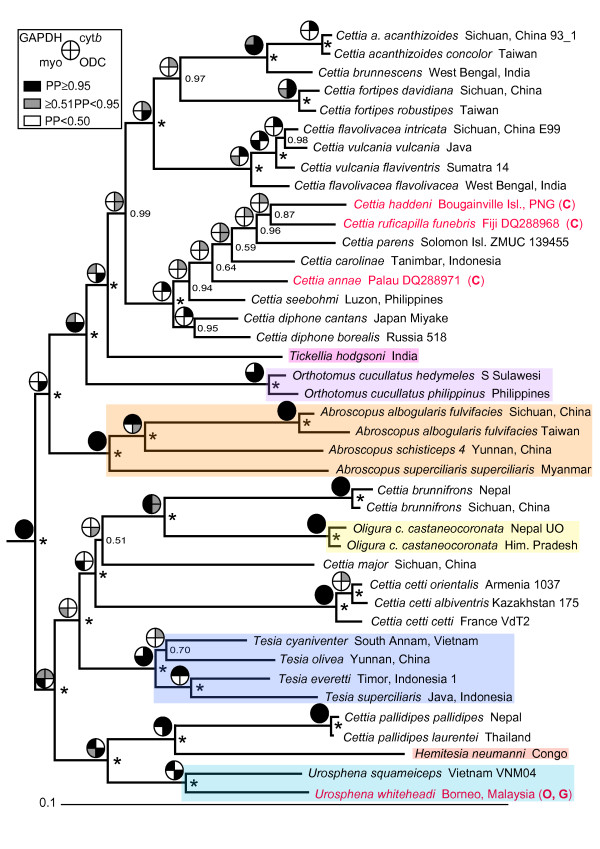
**Tree of Cettiidae based on concatenated sequences, with single-locus analyses superimposed - all species, partitioned analysis**. Majority rule (50%) consensus tree, based on concatenated nuclear ODC, myoglobin and GAPDH and mitochondrial cytochrome *b*, inferred by Bayesian inference (BI), analysed in four partitions. All species, but only a small number of subspecies, were included. Generic affinity according to traditional taxonomy [12] is indicated by different colour shadings. The three species for which only cytochrome *b *(C) is available, and the single species for which only ODC (O) and GAPDH (G) are available, are in red. Posterior probabilities are indicated at the nodes; an asterisk represents posterior probability 1.00. The outgroups (*Alauda arvensis *and *Mirafra javanica *in Alaudidae and *Orthotomus sepium*, *O. sutorius *and *Prinia familiaris *in Cisticolidae) have been pruned from the tree. Pie charts at nodes denote support in single-locus analyses (see explanation in upper left corner; see also Additional files 1, 2, 3 and 4).

The partitioned, mixed-model MrBayes analysis of the concatenated data including all sequences is shown in Figure 1, and a tree including a smaller number of subspecies/individuals, with the results from single-locus analyses indicated, is shown in Figure 2. Two main clades (A and B) are inferred, although clade A is not unanimously strongly supported (PP 1.00, ML 77%, MP 51%). Clade A contains 12 of the 16 species of *Cettia *(clade E), the monotypic genus *Tickellia*, *Orthotomus cucullatus *and the three species of *Abroscopus*. Clade B comprises the remaining four species of *Cettia*, the four species of *Tesia*, the two species of *Urosphena *and the monotypic genera *Oligura *and *Hemitesia*. In clade A, all four genera are monophyletic, with *Cettia *(clade E) being divided into a mostly continental Asian clade (H) and a mainly Pacific islands clade (I). Clade B is split into two main clades, one (F) comprising three species of *Cettia*, *Oligura *and *Tesia*, and the other (G) containing one species of *Cettia*, *Urosphena *and *Hemitesia*.

*C. vulcania *and *C. diphone *are suggested to be non-monophyletic, although the support for this is weak. Moreover, the monophyly of *C. flavolivacea *is poorly supported, and one of its subspecies, *intricata*, is inferred to be non-monophyletic. Monophyly is unsupported for a few subspecies of some other species. Deep intraspecific divergences are revealed within *C. flavolivacea*, *C. fortipes *and *C. cetti*.

The topology of the tree estimated from the unpartitioned, single-model data differs in several minor aspects from the one in Figure 1 (Additional file 5), although no incongruence has PP ≥ 0.95 in both analyses. Likewise, the tree resulting from the partitioned, mixed-model analysis containing only taxa for which all loci are available (Additional file 6) differs slightly from the tree in Figure 1. Notably, in the absence of the three Pacific islands species for which only cyt*b *is available (highlighted in red in Figure 1), *Cettia carolinae *and *C. parens *form a strongly supported clade (PP 1.00), as do *Cettia diphone borealis *and *C. seebohmi *(PP 1.00).

### *BEAST analyses

**Figure 3:**
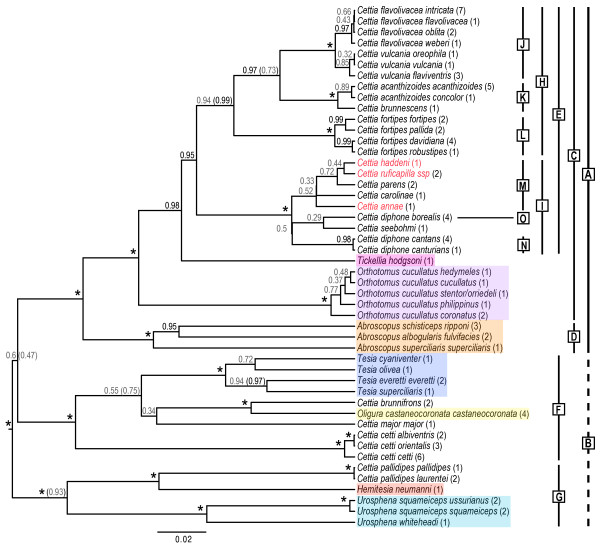
**Phylogeny of Cettiidae based on species tree analysis**. Majority rule (50%) consensus tree, based on nuclear ODC, myoglobin and GAPDH and mitochondrial cytochrome *b*, inferred by *BEAST. All available sequences were included; individuals traditionally classified as the same subspecies were grouped *a priori*, but were not predefined as belonging to the same species; all loci had independent substitution models; and a relaxed clock prior was applied ("Subspecies jModelTest relaxed"). Generic affinity according to traditional taxonomy [12] is indicated by different colour shadings. Labelled bars denote clades discussed in text. The three species for which only cytochrome *b *is available are in red. Values in parentheses after names are the number of individuals included. Posterior probabilities (PPs) are indicated at the nodes; * means PP 1.00; PPs < 0.95 are in grey font; PPs in parentheses are from an analysis where subspecies of a species were predefined as being conspecific, all else being equal ("jModelTest relaxed"), when PPs deviate by > 0.10.

The tree based on the "Subspecies jModelTest relaxed" model is shown in Figure 3. Although this is slightly inferior to the "jModelTest relaxed" tree according to the BF analysis (Table 2), there are no topological conflicts with PP ≥ 0.95 in both trees. Furthermore, the former tree contains more information, as the subspecies are shown. The differences between these two trees are indicated in Figure 3. The tree resulting from the analysis containing only taxa for which all loci are available ("Full jModelTest relaxed"; Additional file 7) is identical to the tree in Figure 3 with respect to relationships (except for the excluded species), although relative branch lengths and some support values vary (latter both higher and lower).

The topology of the *BEAST tree agrees well with the MrBayes tree, with two exceptions: (1) *BEAST does not infer the basal split between clades A and B, and instead identifies clade F as sister to clade A, although with low statistical support. This is the case in all *BEAST analyses with a relaxed clock model, whereas all *BEAST analyses using a strict clock model recover clade B, although with rather low support (PP mean 0.80 ± 0.06; not shown). (2) Both *C. flavolivacea *and *C. vulcania *are monophyletic.

### Indels

Several indels in the nuclear introns support certain clades (Figure 1). It is also noteworthy that some indels are homoplasious (as remarked in footnotes). Interestingly, this concerns a deletion of eight base pairs in the GAPDH alignment, which is found in clade J and also in *Cettia cetti *and *C. fortipes fortipes *from west Myanmar (indicated by 2 in Figure 1), and a deletion of four base pairs in the ODC alignment in both clade H and *Tickellia hodgsoni *(indicated by 3 in Figure 1).

## Discussion

### Model selection and comparison of methods

With respect to the MrBayes analyses including all sequences, Bayes Factors strongly favour the partitioned, mixed-model analysis over the unpartitioned, single-model analysis. It could be argued that mixed-model analyses are inherently superior to single-model analyses of concatenated data (e.g. [23,24]), especially in cases where different loci have markedly different phylogenetic signal. In the present study, the mitochondrial cyt*b *is considerably more informative than the three nuclear loci, and in the single-model analysis of the concatenated data cyt*b *obviously influences the topology more than the nuclear loci (cf. e.g. the *C. flavolivacea-C. vulcania *and *C. fortipes *clades in Figures 1 and 2 and Additional File 2). In contrast, the partitioned, mixed-model analysis produces a more balanced estimate of the relationships.

As concatenation has been shown in simulations to be statistically inconsistent and to positively select the wrong species tree under certain circumstances (e.g. [25-28]), species tree approaches, such as *BEAST, might be expected to provide a better estimate of the phylogeny of Cettiidae than concatenation. The superiority of *BEAST over concatenation in estimating the species tree topology has been demonstrated using simulated data [20]. However, in the present study there are no strongly supported incongruences between different single-locus analyses and, as expected, good agreement exists between the trees reconstructed via the species tree approach and concatenation. The conflicts between the topology estimates of the concatenated MrBayes analysis and the *BEAST are restricted to nearby branches. We could not detect any signs of the latter method receiving additional signal from the likelihood function of gene trees given a species tree (cf. [27,29-32]). It should be noted, however, that nearly half the species in the present study include only one individual, thereby not taking full advantage of the multispecies coalescent model of *BEAST.

### Interspecific relationships

The extremely high degree of non-monophyly in the genus *Cettia *suggested by our data is strongly supported. This level of non-monophyly was completely unexpected, and is likely to be one of the most remarkable examples of misinterpreted relationships in an avian genus.

**Figure 4:**
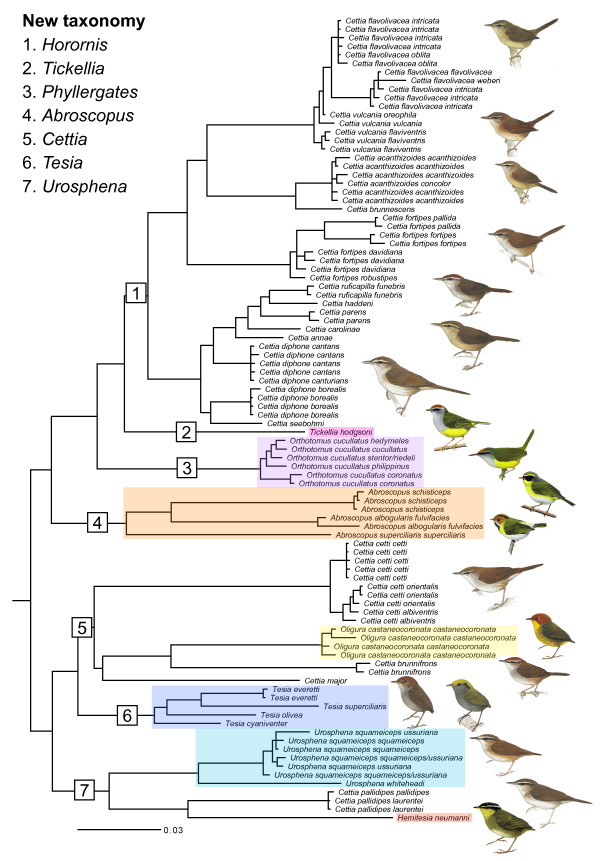
**Revised classification**. Same tree as in Figure 1, but with illustrations of a selection of the species, and the revised taxonomy proposed here. Illustrations by Brian Small (*Cettia*, *Oligura*, *Tesia*, *Urosphena*) from Kennerley & Pearson [16] and by Ren Hathway (*Orthotomus*), Brian Small (*Hemitesia*) and Jan Wilczur (*Abroscopus*, *Tickellia*) from Bairlein *et al*. [13].

Overall, the tree is well supported. However, the relationships among the most basal nodes are somewhat uncertain. The split into clades A and B is not strongly supported in all of the analyses. The inclusion of *Abroscopus *(clade D) in clade A is strongly supported in all Bayesian analyses, but less well supported in the ML and unsupported in the MP bootstraps, and is only inferred by one of the single-locus analyses (ODC). The somewhat ambiguous results, in combination with the aberrant morphology of the species in *Abroscopus *compared to the other taxa in Cettiidae (cf. [13] and Figure 4), suggest that more data are needed to corroborate clade A. Clade B is well supported by the concatenation analyses (except for modest MP bootstrap support, 67%) and is inferred by three single-locus analyses (one with PP ≥ 0.95). However, it is not recovered in most of the species tree analyses, although support for the alternative topologies is poor. More data are needed to evaluate this.

The inclusion of *Orthotomus cucullatus *and the monotypic genus *Tickellia *in clade C is unexpected from a morphological point of view (cf. [13] and Figure 4). However, this is strongly supported in all analyses, including three single-locus analyses as well as by two apparently synapomorphic deletions, one in the ODC and one in the GAPDH alignments.

Clades H and I are strongly supported in the species tree and concatenation analyses; clade H is inferred, with strong support, in two and clade I in one of the single-locus analyses. Within clade H, the relative positions of clades J-L are uncertain, as both the topology and support vary among the analyses (cf. Figures 1, 2 and 3).

Clade M has very low PP in both the species tree and concatenation analyses including all taxa (0.52 and 0.85, respectively), and low bootstrap support. However, for three of the five species in this clade, only cyt*b *is available. In contrast, in analyses comprising only species for which all loci are available, *C. carolinae *and *C. parens *form a clade with PP 0.82 in *BEAST and 1.00 in MrBayes. A close relationship between *C. parens *and *C. ruficapilla *has previously been assumed based on morphological similarity, and these two have been placed in their own genus, *Vitia*, whereas *C. annae *has been placed in the monotypic genus *Psamathia *(e.g. [4]). Orenstein & Pratt [33] concluded, based on song and morphological characteristics, that these three species were closely related to *C. diphone *(including *C. seebohmi*, which was at the time considered conspecific with *C. diphone*; *C. carolinae *and *C. haddeni *had not yet been described). Using cyt*b *sequence data for a small number of *Cettia *and one *Urosphena *species, LeCroy and Barker [15] inferred a close relationship among *C. haddeni*, *C. ruficapilla*, *C. parens *and *C. annae *(*C. carolinae *was not included). Clade M makes sense also from a biogeographical perspective, as the species in this clade, together with *C. seebohmi*, are the only members of Cettiidae occurring on southwest Pacific islands [13,16]. This has been suggested previously [[15,33]; latter excluding the two species not described at the time]. Also in agreement with the distributional pattern, the three easternmost species, *C. haddeni*, *C. parens *and *C. ruficapilla*, form a clade (PP 0.95 in MrBayes and 0.72 in *BEAST), although the relationships among these are uncertain.

Clades F and G are well supported in species tree and concatenation analyses. The relationships within clade G are robust, although within clade F they are highly uncertain, except for the sister relationships between *Oligura castaneocoronata *and *Cettia brunnifrons *and between *Tesia everetti *and *T. superciliaris*, which are both well supported. Irestedt *et al*. [3] found the monotypic genus *Hemitesia *to be sister to *Urosphena squameiceps*, although they did not include *Cettia pallidipes *in their analysis. The only missing species in Cettiidae, *Urosphena subulata*, is most likely to be closely related to the two other *Urosphena*, which it closely resembles in morphology and vocalizations [8,13,16].

### Intraspecific relationships

Olsson *et al*. [19] concluded, based on congruence of cyt*b *and myoglobin gene trees, that *Cettia vulcania *is nested within *C. flavolivacea*. This is contradicted by the present study, which comprises a larger number of loci and samples (including all of the samples from Olsson *et al*. [19]). In contrast to the previous study, *C. flavolivacea *is here inferred to be monophyletic in both the *BEAST and MrBayes analyses, whereas *C. vulcania *is non-monophyletic in the MrBayes tree. However, neither of these relationships is strongly supported by the data. Moreover, all eight samples of *C. flavolivacea *have a three base pairs deletion in the ODC alignment that is not shared with any other taxon, further supporting the monophyly of *C. flavolivacea*. In contrast, the parsimony bootstrap strongly supports the non-monophyly of *C. flavolivacea *found by Olsson *et al*. [19]. This is in agreement with, and presumably heavily influenced by, the cyt*b *data.

The MrBayes tree infers deep divergences between two main *C. flavolivacea *clades. In this tree, as well as in the cyt*b *tree, Sichuan and Yunnan *intricata *are in different, rather deeply divergent clades, the former together with Vietnamese *oblita *and the latter with Himalayan *flavolivacea *and west Myanmar *weberi*. In contrast, the *BEAST tree infers only marginal differences between the four *C. flavolivacea *subspecies. The monophyly of *C. flavolivacea intricata *in the *BEAST phylogeny is illusory, as this taxon was constrained to be monophyletic in this analysis, as *BEAST requires all predefined taxa to be monophyletic. This is a limitation and drawback of *BEAST (as it is also for two other multispecies coalescent methods, BEST and STEM, as remarked by Leaché & Rannala [34]). A promising solution to the problem of specifying species delimitation *a priori *has recently been suggested [35]. The single-locus nuclear trees offer no solution, as they are poorly resolved/supported. In conclusion, more data, including unsampled subspecies, are needed to resolve the relationships in the *C. flavolivacea*-*C. vulcania *complex.

With respect to *Cettia fortipes*, the trees resulting from the *BEAST and concatenation analyses differ markedly from the cyt*b *tree. The parsimony bootstrap of the concatenated data supports the same topology as the cyt*b *tree, presumably heavily influenced by the cyt*b *data. Single-locus analyses of the nuclear loci are inconclusive. More data are needed to resolve these relationships.

In the multilocus and cyt*b *trees, *Cettia diphone *is separated into two divergent clades, one comprising the Japanese subspecies *cantans *and Chinese *canturians *(N) and the other representing the northern subspecies *borealis *(O). In both multilocus BI trees including all taxa, *borealis *is sister to *C. seebohmi*, although with low support. In the MrBayes analysis excluding the three species for which only cyt*b *is available, this relationship receives PP 1.00 (no comparable *BEAST analysis was performed). This topology is not supported in the ML and MP bootstrap analyses. More data are required, including sequences of the missing subspecies.

The samples of *Cettia cetti *are separated into two rather divergent, well-supported clades, representing western and eastern populations, respectively.

### Unexpected relationships due to complex morphological evolution

Cettiidae comprises a mixture of taxa that had not been considered closely related before the advent of DNA sequence analyses. Alström *et al*. [1] showed, based on cyt*b *and myoglobin sequence data, that two species of *Cettia*, and one species each of *Urosphena*, *Tesia*, *Abroscopus *and *Tickellia *and *Orthotomus cucullatus *formed a clade, well separated from a broad selection of other passerines. *Hemitesia *was later shown to be part of this clade [3]. Morphological support of this unexpected group was provided by the fact that all of these taxa have 10 rectrices (eight in the extremely short-tailed *Tesia*), in contrast to 12 in most other passerine birds [1,3]. The present study corroborates these results, and reiterates the complex morphological evolution within this group (cf. Figure 4), which has misled earlier taxonomists (e.g. [4-13,16]). The rather colourful and strikingly patterned *Tickellia*, *Orthotomus cucullatus*, *Abroscopus *(notably *A. schisticeps *and *A. albogularis*), *Oligura*, *Hemitesia *and one, especially, of the four species of *Tesia *are scattered across the phylogeny among the dull and nondescript *Cettia *and *Urosphena *(three species of *Tesia *are also rather dull in coloration). Moreover, species with extremely short tails appear on three separate branches. This suggests instances of parallel evolution, as well as cases of both highly conserved morphological evolution and strong morphological divergence. A detailed investigation of this is beyond the scope of this paper.

In the case of *Cettia *warblers, the overall resemblance in plumage and structure has been taken as evidence of close relationship among the different species without any cladistic analysis of these characters. The present study strongly underscores the well-known but still often neglected problem of defining groups based on overall morphological similarity (although also molecular characters have been suggested to be essentially "phenetic"; e.g. [36], and comments in [37]).

### Taxonomic implications - genus level

Based on morphological characteristics, the traditional genus *Cettia *has been divided into three subgenera: *Cettia *(containing *C. cetti*), *Urosphena *(containing the three current *Urosphena *and *C. pallidipes*), and *Horeites *(remaining mainland Asian species and *C. seebohmi*); *C. ruficapilla *and *C. parens *were placed in *Vitia *and *C. annae *in *Psamathia*, although it was noted that these genera were closely related to *Cettia *[4]. Watson *et al*. [7] recognised the subgenera *Cettia *and *Horeites *(latter including *Vitia *and *Psamathia*), but treated *Urosphena *as a separate genus. Except for the monotypic subgenus *Cettia *and the subgenus *Urosphena *(including also *Hemitesia*), none of these taxa is supported in the present study.

The generic affiliation of several of the species in clade B has varied over the years. The monotypic genus *Oligura *has frequently been synonymised with *Tesia *(e.g. [4,5,8,10,11]), although the present study strongly supports a closer relationship with at least one species of *Cettia *(*C. brunnifrons*) than with *Tesia*. Moreover, *Tesia everetti *and *Cettia pallidipes *had been placed in *Urosphena *(e.g. [7]), until King [8] suggested, based on structural, behavioural and song characteristics, that the former should be moved to *Tesia *and the latter to *Cettia*. The transfer of *T. everetti *from *Urosphena *to *Tesia *and the removal of *C. pallidipes *from *Urosphena *are corroborated by our data, although the latter's position in *Cettia *is not supported. The genus *Urosphena *has been subsumed in *Cettia *(e.g. [4,6,38], which, based on the current circumscription of *Cettia*, is not supported by the molecular data.

*Orthotomus cucullatus *has been shown to belong in Cettiidae [1,39], and this is strongly supported here. It is also shown here for the first time that the type species of *Orthotomus*, i.e. *O. sepium*, is closely related to *Orthotomus sutorius *(in the family Cisticolidae; [1,39]), and hence not a close relative of *O. cucullatus*. This calls for a change of generic affiliation of *O. cucullatus *(see below).

### Taxonomic implications - species level

Olsson *et al*. [19] recommended, based on cyt*b *and myoglobin sequence data, that the name *Cettia flavolivacea *be restricted to the subspecies *flavolivacea *and *weberi*, whereas the subspecies *intricata *and *oblita *be placed in *C. vulcania*. This proposal is contradicted by some of the data in the present study, although the conflict between different analyses precludes a firm taxonomic view. Kennerley & Pearson [16] disputed the findings by Olsson *et al*. [19] based on plumage characteristics. A more comprehensive study, based on a larger number of loci and including the single missing subspecies of *C. flavolivacea *and the five missing subspecies of *C. vulcania*, as well as morphology and vocalizations, is warranted.

The treatment of *C. vulcania *as conspecific with *C. fortipes *[4,40] or as forming a superspecies with *C. fortipes *[7] has previously been rejected [19]. The present study corroborates the rejection of both treatments.

Based on morphological and vocal differences [16] or without providing any justification [5,9], *C. diphone *is often split into two allopatric species, *C. diphone sensu stricto *in Japan, South Korea and on Sakhalin Island (Russia), and *C. canturians *in continental East Asia. The present study supports a division into two distinct clades (N and O), which might even be non-sisters. However, these clades do not conform to the proposed circumscription of the two species, as our single sample of the subspecies *canturians *is in the *C. diphone sensu stricto *clade (N), i.e. in a different clade compared to its putative closest relative, the subspecies *borealis *(clade O). A more comprehensive sampling will be needed, in combination with a thorough analysis of vocalizations, to evaluate the taxonomy of the *Cettia diphone *complex.

*Cettia seebohmi *has often been treated as a subspecies of *C. diphone sensu lato *(e.g. [4,7,11]), although it has also been considered to be a separate species based on alleged differences in song and lack of the pronounced sexual size dimorphism of *C. diphone*/*C. canturians *[10,12]. Hamao *et al*. [17] compared songs and cyt*b *sequences of *C. seebohmi*, *C. diphone borealis *and *C. diphone cantans*, and concluded that *C. seebohmi *was sufficiently distinct to be recognised as a separate species. The present study lends further support to this conclusion.

This study suggests that *Cettia fortipes *might be better treated as three different species, and that *Cettia cetti *might be treated as two species. Detailed studies of these complexes, including vocalizations, are needed.

### Revised classification

The traditional classification (e.g. [6,7,9,12,13]) is obviously at odds with the results of the present study, and needs to be revised. We propose a revised taxonomy that is shown in Figure 4 and Table 3 (cf. also Table 4, with authors and type species). The recognition of *Tickellia *(comprising *T. hodgsoni*) and *Phyllergates *(comprising *P. cucullatus*) as monotypic genera rather than including them in *Horornis *acknowledges their unique morphology in relation to *Horornis*. The same applies (even more) to the genus *Abroscopus*, and also takes into account the fact that its exact position in the tree is considered somewhat uncertain.

**Table 3:** Taxonomy

Traditional taxonomy	Revised taxonomy
*Abroscopus albogularis *(F. Moore, 1854)	*Abroscopus albogularis *(F. Moore, 1854)
*Abroscopus schisticeps *(J.E. & G.R. Gray, 1846)	*Abroscopus schisticeps *(J.E. & G.R. Gray, 1846)
*Abroscopus superciliaris *(Blyth, 1859)	*Abroscopus superciliaris *(Blyth, 1859)
*Cettia acanthizoides *(J. Verreaux, 1871)	***Horornis acanthizoides ***(J. Verreaux, 1871)
*Cettia annae *(Hartlaub & Finsch, 1868)	***Horornis annae ***(Hartlaub & Finsch, 1868)
*Cettia brunnescens *(Hume, 1872)^a^	***Horornis brunnescens ***(Hume, 1872)^a^
*Cettia brunnifrons *(Hodgson, 1845)	*Cettia brunnifrons *(Hodgson, 1845)
*Cettia carolinae *Rozendaal, 1987	***Horornis carolinae ***(Rozendaal, 1987)
*Cettia cetti *(Temminck, 1820)	*Cettia cetti *(Temminck, 1820)
*Cettia diphone *(Kittlitz, 1830)	***Horornis diphone ***(Kittlitz, 1830)
*Cettia flavolivacea *(Blyth, 1845)	***Horornis flavolivaceus ***(Blyth, 1845)
*Cettia fortipes *(Hodgson, 1845)	***Horornis fortipes ***Hodgson, 1845
*Cettia haddeni *LeCroy & Barker, 2006	***Horornis haddeni ***(LeCroy & Barker, 2006)
*Cettia major *(Moore, 1854)	*Cettia major *(Moore, 1854)
*Cettia pallidipes *(Blanford, 1872)	***Urosphena pallidipes ***(Blanford, 1872)
*Cettia parens *(Mayr, 1935)	***Horornis parens ***(Mayr, 1935)
*Cettia ruficapilla *(E.P. Ramsay, 1876)	***Horornis ruficapilla ***(E.P. Ramsay, 1876)
*Cettia seebohmi *Ogilvie-Grant, 1894	***Horornis seebohmi ***Ogilvie-Grant, 1894
*Cettia vulcania *(Blyth, 1870)	***Horornis vulcanius ***(Blyth, 1870)
*Hemitesia neumanni *(Rothschild, 1908)	***Urosphena neumanni ***(Rothschild, 1908)
*Oligura castaneocoronata *(E. Burton, 1836)	***Cettia castaneocoronata ***(E. Burton, 1836)
*Orthotomus cucullatus *Temminck, 1836	***Phyllergates cucullatus ***(Temminck, 1836)
*Tesia cyaniventer *Hodgson, 1837	*Tesia cyaniventer *Hodgson, 1837
*Tesia everetti *(E. Hartert, 1897)	*Tesia everetti *(E. Hartert, 1897)
*Tesia olivea *(McClelland, 1840)	*Tesia olivea *(McClelland, 1840)
*Tesia superciliaris *(Bonaparte, 1850)	*Tesia superciliaris *(Bonaparte, 1850)
*Tickellia hodgsoni *(F. Moore, 1854)	*Tickellia hodgsoni *(F. Moore, 1854)
*Urosphena squameiceps *(Swinhoe, 1863)	*Urosphena squameiceps *(Swinhoe, 1863)
*Urosphena subulata *(Sharpe, 1884)^b^	*Urosphena subulata *(Sharpe, 1884)^b^
*Urosphena whiteheadi *(Sharpe, 1888)	*Urosphena whiteheadi *(Sharpe, 1888)

Traditional (mainly following Dickinson [12]) and revised taxonomy proposed here (in alphabetical order based on traditional taxonomy). The proposed changes are in bold.

^a^Treated as subspecies of *C. acanthizoides *by Dickinson [12], but split by Alström *et al*. [18]. ^b^Not included in the present study, but tentatively placed in *Urosphena *due to strong morphological and vocal similarity with *U. squameiceps *[8,13,16].

**Table 4:** Authors and type species

*Abroscopus *E.C.S. Baker, 1930 Type species: *Abroscopus superciliaris*
*Cettia *Bonaparte, 1834 Type species: *Cettia cetti*
*Hemitesia *Chapin, 1948 Type species: *Hemitesia neumanni*
*Horornis *Hodgson, 1845 Type species: *Cettia fortipes*
*Oligura *Hodgson, 1844 Type species: *Oligura castaneocoronata*
*Orthotomus *Horsfield, 1821 Type species: *Orthotomus sepium*
*Phyllergates *Sharpe, 1883 Type species: *Orthotomus cucullatus*
*Tesia *Hodgson, 1837 Type species: *Tesia cyaniventer*
*Tickellia *Blyth, 1861 Type species: *Tickellia hodgsoni*
*Urosphena *Swinhoe, 1877 Type species: *Urosphena squameiceps*

Authors and type species of the genera used by Dickinson [12] and in the revised taxonomy proposed here (in alphabetical order of genera). Names of type species follow [12].

Although *Phyllergates cucullatus *has been known to belong in Cettiidae for some time [1,39], the type species of *Orthotomus*, i.e. *O. sepium*, has not previously been included in a phylogenetic analysis. Accordingly, it is only now that it is confirmed that the genus name *Orthotomus *does not apply to the clade to which *cucullatus *belongs. As only a minority of the species in the genus *Orthotomus *have been studied phylogenetically, it is possible that more species will be included in *Phyllergates*.

The circumscription of *Cettia*, as proposed here, is not entirely satisfactory, as this clade is not inferred by *BEAST, is not strongly supported in all of the concatenation analyses, and is only recovered in one single-locus analysis (though supported by a unique deletion in the myo alignment). Inclusion of *Tesia *in *Cettia *might have been more appropriate based on the molecular data, although we tentatively prefer to treat *Tesia *as a separate genus, acknowledging that it is a morphologically well-defined group of long standing. An alternative would be to recognise a monotypic genus *Cettia *(including *cetti*), propose a new generic name for *major*, and place *brunnifrons *and *castaneocoronata *in *Oligura*.

The genus *Urosphena*, as defined here, is morphologically and vocally heterogeneous (cf. [8,13,16]), although it is well supported by the molecular data. An alternative would be to restrict *Urosphena *to the morphologically and vocally well defined group comprising *U. squameiceps*, *U. whiteheadi *and *U. subulata *([8,13,16]; latter not included in present study), and either place both *U. neumanni *and *U. pallidipes *in the genus *Hemitesia *or recognise a monotypic genus *Hemitesia *(comprising *U. neumanni*) and propose a new generic name for *U. pallidipes*.

Future studies are needed to evaluate the taxonomy of several of the species that have been shown here to have pronounced intraspecific genetic divergence.

## Conclusions

The molecular phylogeny presented here is highly inconsistent with the traditional, morphology-based classification. There are probably few equally striking examples in an avian genus of mismatch between deductions made on morphological evidence and insights resulting from molecular analysis. The phylogeny suggests that morphological evolution within Cettiidae has been extremely complex, with examples of highly conserved phenotypes as well as dramatic morphological divergence and instances of parallel evolution. This unexpected intricacy has evidently misguided earlier taxonomists. A revised taxonomy is proposed.

## Methods

### Study group

Species level taxonomy follows Dickinson [12], except for the recognition of *Cettia brunnescens *as a separate species from *C. acanthizoides *[18]. In total, 48 taxa in the family Cettiidae (*sensu *Alström *et al*. [1]) were included. This comprises all recognised species, except the Timor and Babar Islands (Indonesia) endemic *Urosphena subulata*, as well as large number of subspecies (Additional file 8), in total more than 50% of all recognised taxa (cf. [7,9,12,13]). For most taxa, multiple sequences were available, in total 94 ingroup sequences (Additional file 8). As outgroups in the MrBayes, maximum likelihood and parsimony analyses (see below), three species belonging to the family Cisticolidae (*Orthotomus sutorius*, *O. sepium*, *Prinia familiaris*) were chosen, as this family is closely related to Cettiidae [1,2], and two representatives from the slightly more distantly related Alaudidae (*Alauda arvensis*, *Mirafra javanica*) [1,2].

### DNA extraction and sequencing

DNA was extracted from blood, feathers or muscle using QIA Quick DNEasy Kit (Qiagen, Inc) according to the manufacturer's instruction, but with 30 μl 0.1% DTT added to the initial incubation step of the extraction of feathers. We sequenced four loci: the main part of the mitochondrial cytochrome *b *gene and part of the flanking tRNA-Thr (hereafter cyt*b*); the nuclear ornithine decarboxylase introns 6 and 7 and exons 7 and parts of 6 and 8 (ODC); the entire nuclear myoglobin intron 2 (myo), and the nuclear glyceraldehyde-3-phosphodehydrogenase intron 11 (GAPDH). Amplification and sequencing of cyt*b *and myo followed the protocols described in Olsson *et al*. [41], of ODC Allen & Omland [42], and of GAPDH Fjeldså *et al*. [43]. Cyt*b *was amplified as one fragment to decrease the risk of amplifying nuclear pseudocopies (e.g. [44]). All new sequences have been deposited in GenBank (Additional file 8).

### Phylogenetic analyses

Sequences were aligned using MegAlign 4.03 in the DNASTAR package (DNAstar Inc.); some manual adjustment was necessary for the non-coding sequences. Gene trees were estimated by Bayesian inference (BI) using MrBayes 3.1.2 [45,46] according to the following: (1) all loci were analysed separately (single-locus analyses); (2) sequences were also concatenated, all loci together. In the multilocus analyses, the data were either (a) partitioned by locus, using rate multipliers to allow different rates for the different partitions [47,48], or (b) unpartitioned, using the same model for the entire dataset. Moreover, partitioned multilocus analyses were also run including(a) all available sequences, i.e. also samples for which only one or two loci were available (with the missing sequences represented by? in the matrix), and (b) only those samples for which all four loci were available.

Appropriate substitution models were determined based on the Bayesian Information Criterion [49] calculated by jModelTest version 0.1.1 [50]. For ODC, posterior probabilities (PPs) were calculated under the general time-reversible (GTR) model [51-53], assuming rate variation across sites according to a discrete gamma distribution with four rate categories (Γ; [54]); for the three other loci the HKY model [55] was selected, for the cyt*b *data also an estimated proportion of invariant sites (I; [56]). For the unpartitioned dataset, the GTR+Γ+I model was selected. Default priors in MrBayes were used. Four incrementally heated Metropolis-coupled MCMC chains with temperature 0.1 or 0.2 were run for 10-50 × 10^6 ^generations and sampled every 1000 generations. Convergence to the stationary distribution of the single chains was inspected using a minimum threshold for the effective sample size. The joint likelihood and other parameter values reported large effective sample sizes (> 200, generally > 1000), and were inspected in Tracer 1.5.0 [57]. The first 25% of the generations were discarded as "burn-in", well after stationarity of chain likelihood values had been established, and the posterior probabilities were calculated from the remaining samples. Good mixing of the MCMC and reproducibility was established by multiple runs from independent starting points. Each analysis was run at least twice, and the topologies and posterior probabilities were compared by eye and by the difference of mean estimates of independent runs within the expected range (±3* Monte Carlo Standard Error [58]).

Integrative species tree estimation was performed using *BEAST [20], where gene trees and species trees are estimated simultaneously. *BEAST uses a multilocus species estimation by the multispecies coalescent for estimating the species tree, and hence can incorporate multilocus data from multiple individuals. Species delimitation has to be defined *a priori *in *BEAST. We followed the default settings and recommendations of *BEAST to set up the models. Nevertheless, we ran analyses under a panel of different models: (1) different substitution models which were either (a) a GTR+Γ+I model for the cyt*b *sequences and HKY for the other sequences (referred to as "GTR"), or (b) the same substitution models per partition as in the MrBayes analysis (referred to as "jModelTest"); (2) these models were combined with either of two clock models, (a) being a strict molecular clock (referred to as "strict"), or (b) a uncorrelated lognormal distributed relaxed clock [59] (referred to as "relaxed"). A piecewise linear population size model with a constant root was used as a prior for the multispecies coalescent and a birth-death model [60] as prior on divergence times. All analyses were run including (a) all available sequences, and (b) only individuals for which all loci were available.

To establish how well each model fit the data, we calculated Bayes Factors (BF; [21,22]) in Tracer 1.5.0 [57] using the harmonic mean as an approximation of the marginal likelihood of a model.

Maximum likelihood bootstrapping (1000 replicates) was performed on the complete dataset in RAxML 7.2.8 [61,62] at the CIPRES Science Gateway [63], using the GTRCAT algorithm for the bootstrapping phase, and GTRGAMMA for the final tree inference (as per default); the dataset was partitioned as in the Bayesian analyses. Parsimony bootstrapping was performed in PAUP* [64] on the complete dataset: heuristic search strategy, 1000 replicates, starting trees obtained by stepwise addition (random addition sequence, 10 replicates), TBR branch swapping, MulTrees option not in effect (only one tree saved per replicate).

Alignments and trees have been deposited in TreeBASE (http://www.treebase.org/treebase-web/home.html; accession http://purl.org/phylo/treebase/phylows/study/TB2:S11953).

## Authors' contributions

PA participated in the design of the study, acquisition of data, analysis and interpretation of data, and drafted the manuscript. SH participated in the analysis and interpretation of data. MG and PGPE participated in the acquisition of data. UO participated in the design of the study, acquisition of data, and analysis and interpretation of data. All authors read and approved the final manuscript.

## Supplementary Material

Additional file 1 **Cytochrome *b *gene tree**. Majority rule (50%) consensus tree of Cettiidae based on mitochondrial cytochrome *b *sequences, inferred by Bayesian inference. All available sequences (including all subspecies) were included. Posterior probabilities are indicated at the nodes; an asterisk represents posterior probability 1.00. Click here for file

Additional file 2 **ODC gene tree**. Majority rule (50%) consensus tree of Cettiidae based on nuclear ornithine decarboxylase introns 6 and 7 and exons 7 and parts of 6 and 8 (ODC) sequences, inferred by Bayesian inference. All available sequences (including all subspecies) were included. Posterior probabilities are indicated at the nodes; an asterisk represents posterior probability 1.00. Click here for file

Additional file 3 **Myoglobin gene tree**. Majority rule (50%) consensus tree of Cettiidae based on nuclear myoglobin intron 2 sequences, inferred by Bayesian inference. All available sequences (including all subspecies) were included. Posterior probabilities are indicated at the nodes; an asterisk represents posterior probability 1.00. Click here for file

Additional file 4 **GAPDH gene tree**. Majority rule (50%) consensus tree of Cettiidae based on nuclear nuclear glyceraldehyde-3-phosphodehydrogenase intron 11 (GAPDH) sequences, inferred by Bayesian inference. All available sequences (including all subspecies) were included. Posterior probabilities are indicated at the nodes; an asterisk represents posterior probability 1.00. Click here for file

Additional file 5 **Tree of Cettiidae based on concatenated sequences - all taxa, unpartitioned analysis**. Majority rule (50%) consensus tree of Cettiidae based on concatenated nuclear ODC, myoglobin and GAPDH and mitochondrial cytochrome b sequences, inferred by unpartitioned Bayesian inference. All available sequences (including all subspecies) were included. Generic affinity according to traditional taxonomy (Dickinson, 2003) indicated by different colour shadings. The three species for which only cytochrome b is available are in red. Posterior probabilities are indicated at the nodes; an asterisk represents posterior probability 1.00. A red § indicates a clade with marked difference compared to partitioned analysis (Figure 1). Click here for file

Additional file 6 **Tree of Cettiidae based on concatenated sequences - complete data, partitioned analysis**. Majority rule (50%) consensus tree of Cettiidae based on concatenated nuclear ODC, myoglobin and GAPDH and mitochondrial cytochrome *b *sequences, inferred by Bayesian inference, analysed in four partitions. Only individuals for which all sequences were available were included (cf. Figure 1). Generic affinity according to traditional taxonomy [12] indicated by different colour shadings. Posterior probabilities are indicated at the nodes; an asterisk represents posterior probability 1.00. The outgroups (*Alauda arvensis *and *Mirafra javanica *in Alaudidae and *Orthotomus sepium*, *O. sutorius *and *Prinia familiaris *in Cisticolidae) have been pruned from the tree. Click here for file

Additional file 7 **Phylogeny of Cettiidae**. Inferred by *BEAST. Only individuals for which all loci were available were included (cf. Figure 3). All loci had independent substitution models; and a relaxed clock prior was applied ("Full jModelTest relaxed"). Generic affinity according to traditional taxonomy [12] is indicated by different colour shadings. Values in parentheses after names are the number of individuals included. Posterior probabilities are indicated at the nodes; an asterisk represents posterior probability 1.00. Click here for file

Additional file 8 **List of samples (in alphabetical order), with GenBank accession numbers**. Click here for file
