# Supplementary figures and images for: Non-monophyly and intricate morphological evolution within the avian family Cettiidae revealed by multilocus analysis of a taxonomically densely sampled dataset
Source: BMC Evol Biol. 2011 Dec 5;11:352. doi: 10.1186/1471-2148-11-352 (PMC3261208; doi:10.1186/1471-2148-11-352)

cytochrome *b*

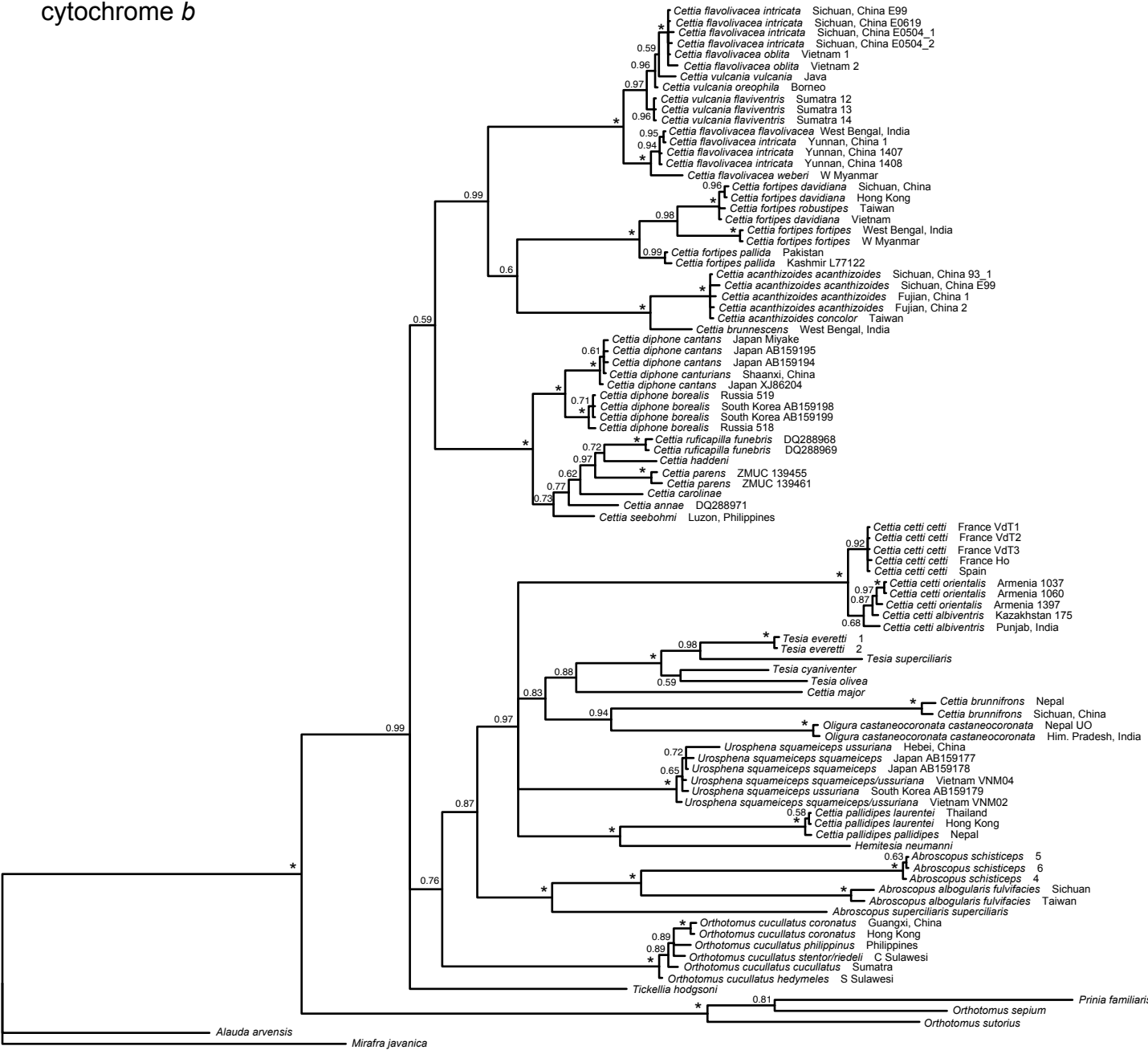

Supplement: Additional file 1 — Cytochrome b gene tree. Majority rule (50%) consensus tree of Cettiidae based on mitochondrial cytochrome b sequences, inferred by Bayesian inference. All available sequences (including all subspecies) were included. Posterior probabilities are indicated at the nodes; an asterisk represents posterior probability 1.00. [file 1471-2148-11-352-S1.PDF]

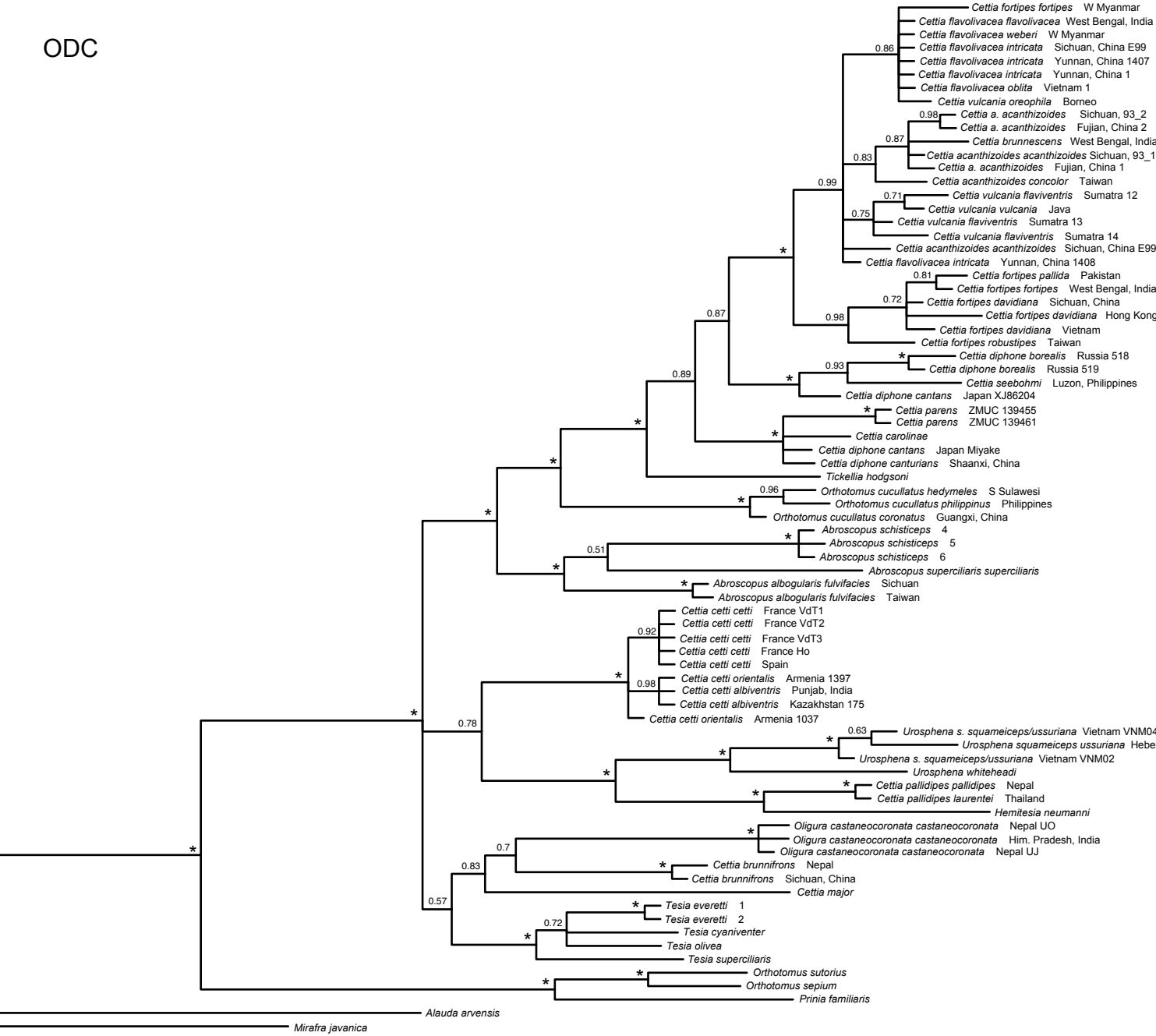

Supplement: Additional file 2 — ODC gene tree. Majority rule (50%) consensus tree of Cettiidae based on nuclear ornithine decarboxylase introns 6 and 7 and exons 7 and parts of 6 and 8 (ODC) sequences, inferred by Bayesian inference. All available sequences (including all subspecies) were included. Posterior probabilities are indicated at the nodes; an asterisk represents posterior probability 1.00. [file 1471-2148-11-352-S2.PDF]

Myoglobin

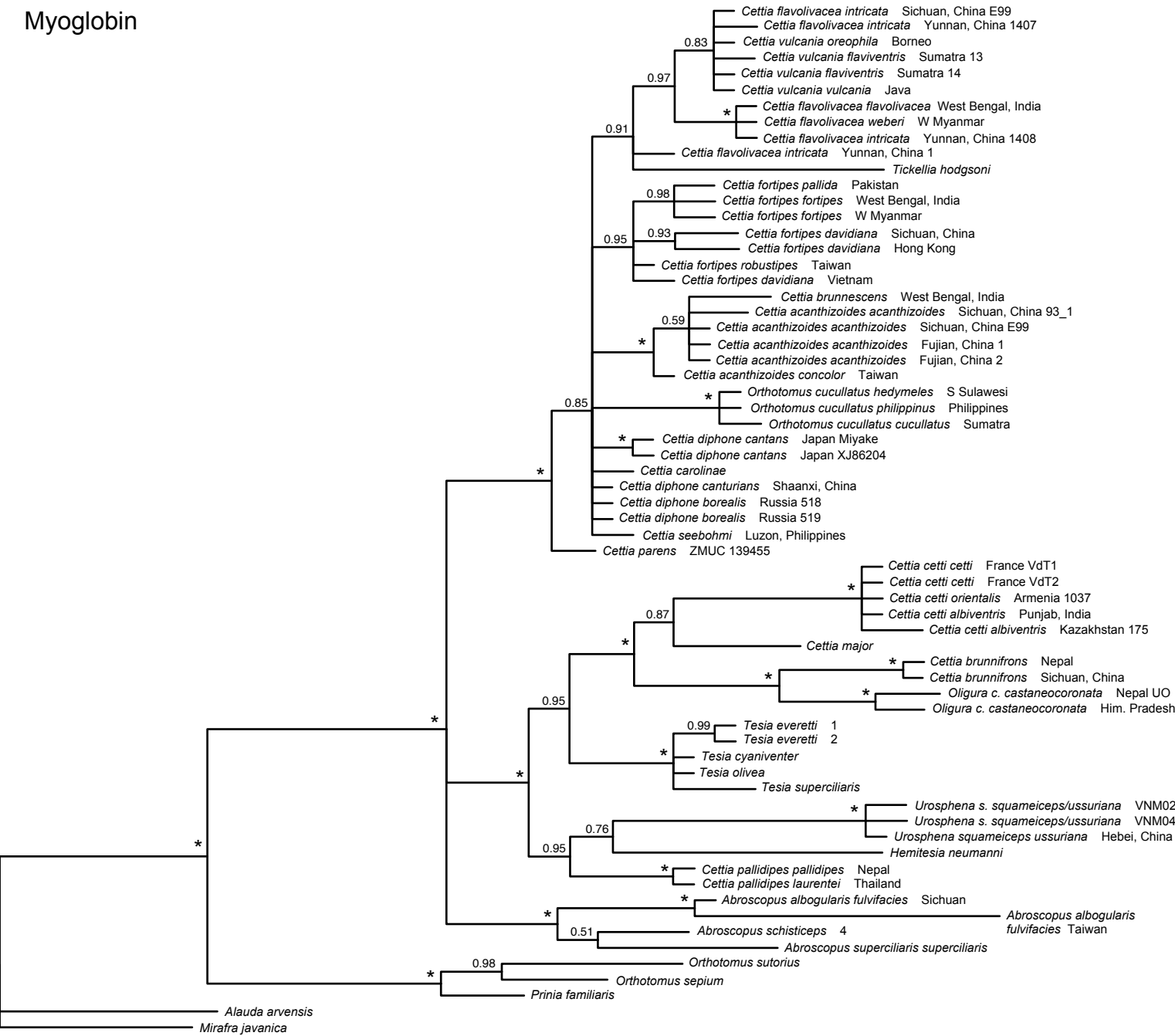

0.0080

Supplement: Additional file 3 — Myoglobin gene tree. Majority rule (50%) consensus tree of Cettiidae based on nuclear myoglobin intron 2 sequences, inferred by Bayesian inference. All available sequences (including all subspecies) were included. Posterior probabilities are indicated at the nodes; an asterisk represents posterior probability 1.00. [file 1471-2148-11-352-S3.PDF]

GAPDH

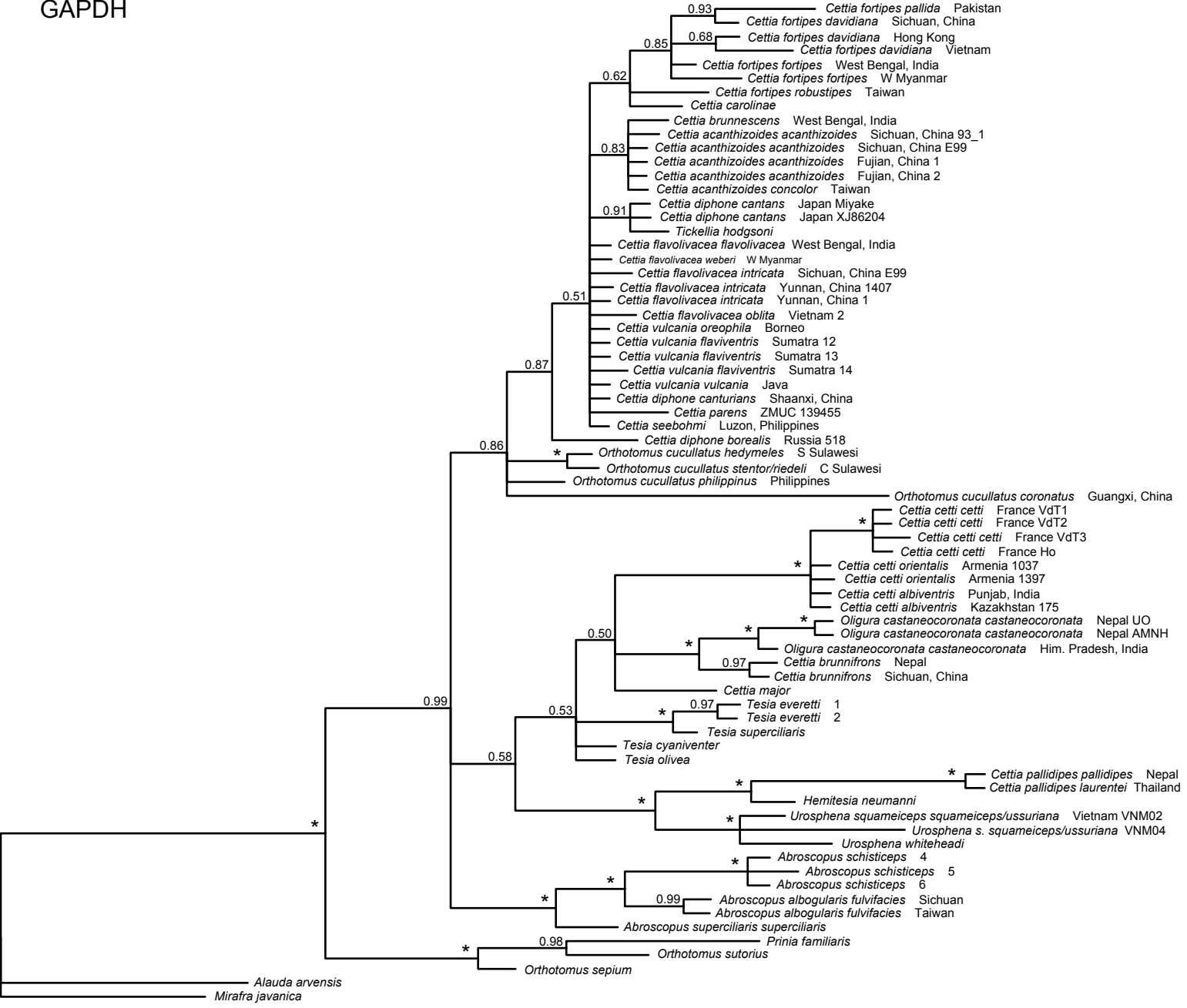

Supplement: Additional file 4 — GAPDH gene tree. Majority rule (50%) consensus tree of Cettiidae based on nuclear nuclear glyceraldehyde-3-phosphodehydrogenase intron 11 (GAPDH) sequences, inferred by Bayesian inference. All available sequences (including all subspecies) were included. Posterior probabilities are indicated at the nodes; an asterisk represents posterior probability 1.00. [file 1471-2148-11-352-S4.PDF]

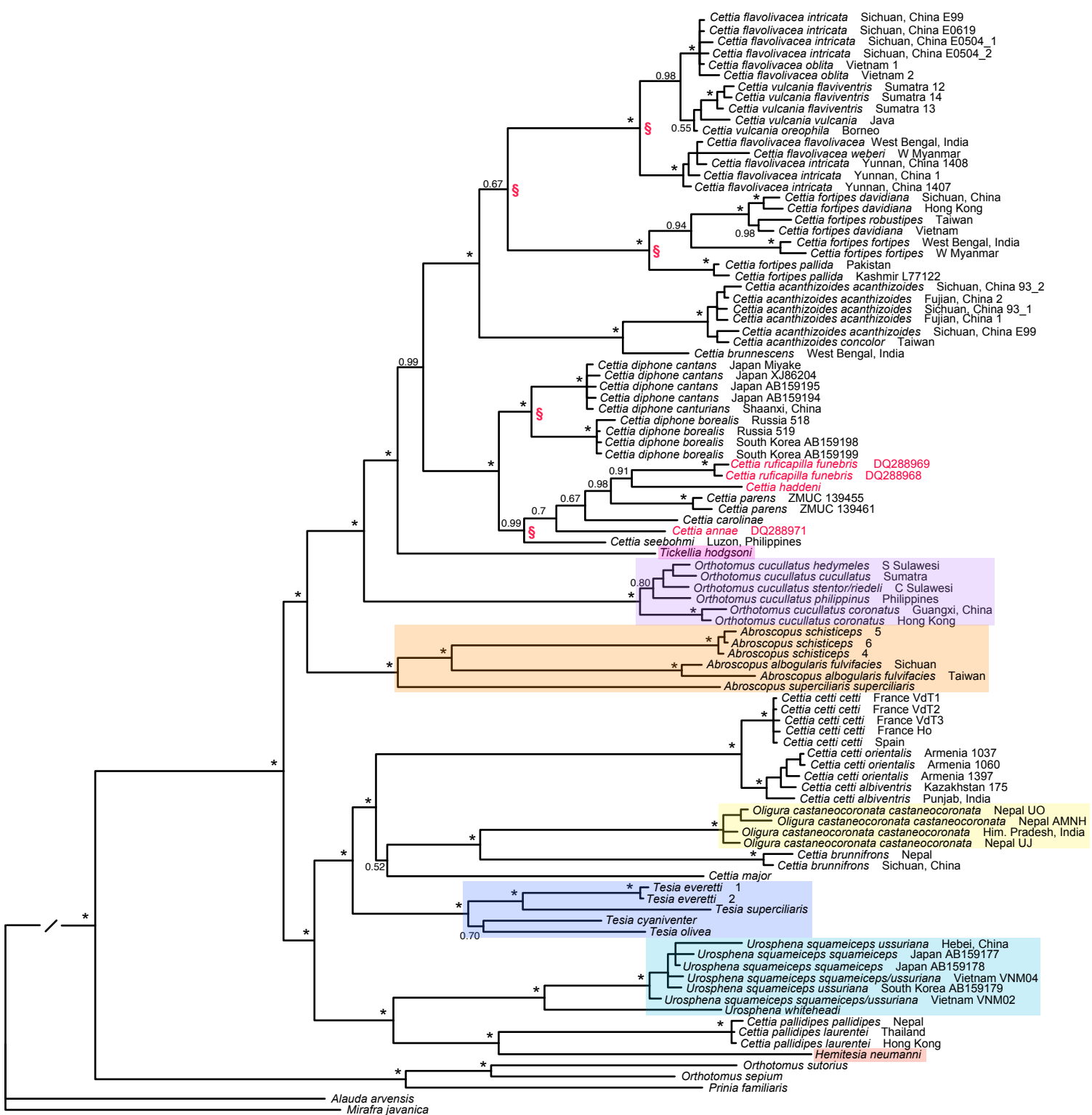

Supplement: Additional file 5 — Tree of Cettiidae based on concatenated sequences - all taxa, unpartitioned analysis. Majority rule (50%) consensus tree of Cettiidae based on concatenated nuclear ODC, myoglobin and GAPDH and mitochondrial cytochrome b sequences, inferred by unpartitioned Bayesian inference. All available sequences (including all subspecies) were included. Generic affinity according to traditional taxonomy (Dickinson, 2003) indicated by different colour shadings. The three species for which only cytochrome b is available are in red. Posterior probabilities are indicated at the nodes; an asterisk represents posterior probability 1.00. A red § indicates a clade with marked difference compared to partitioned analysis (Figure 1). [file 1471-2148-11-352-S5.PDF]

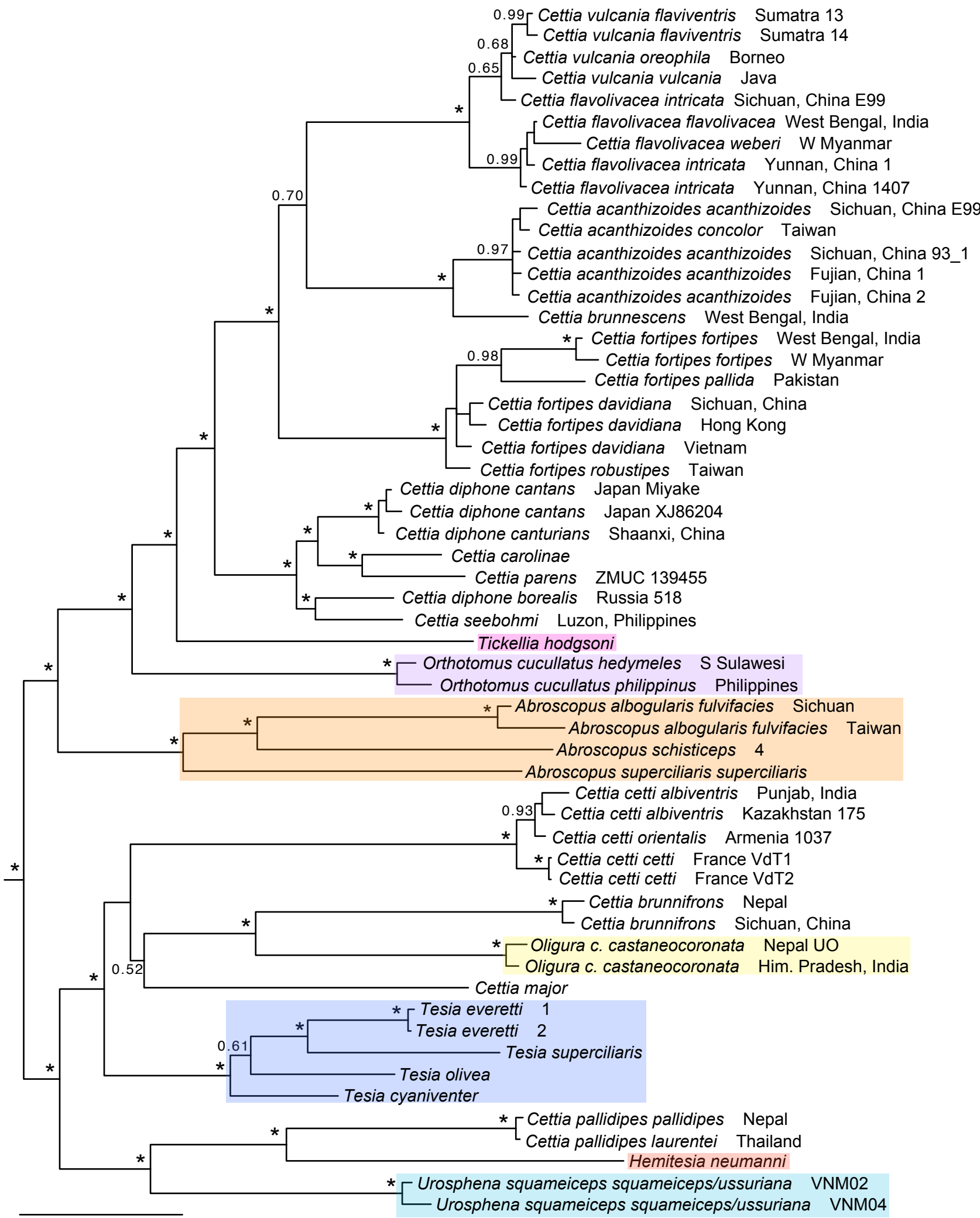

Supplement: Additional file 6 — Tree of Cettiidae based on concatenated sequences - complete data, partitioned analysis. Majority rule (50%) consensus tree of Cettiidae based on concatenated nuclear ODC, myoglobin and GAPDH and mitochondrial cytochrome b sequences, inferred by Bayesian inference, analysed in four partitions. Only individuals for which all sequences were available were included (cf. Figure 1). Generic affinity according to traditional taxonomy [12] indicated by different colour shadings. Posterior probabilities are indicated at the nodes; an asterisk represents posterior probability 1.00. The outgroups (Alauda arvensis and Mirafra javanica in Alaudidae and Orthotomus sepium, O. sutorius and Prinia familiaris in Cisticolidae) have been pruned from the tree. [file 1471-2148-11-352-S6.PDF]

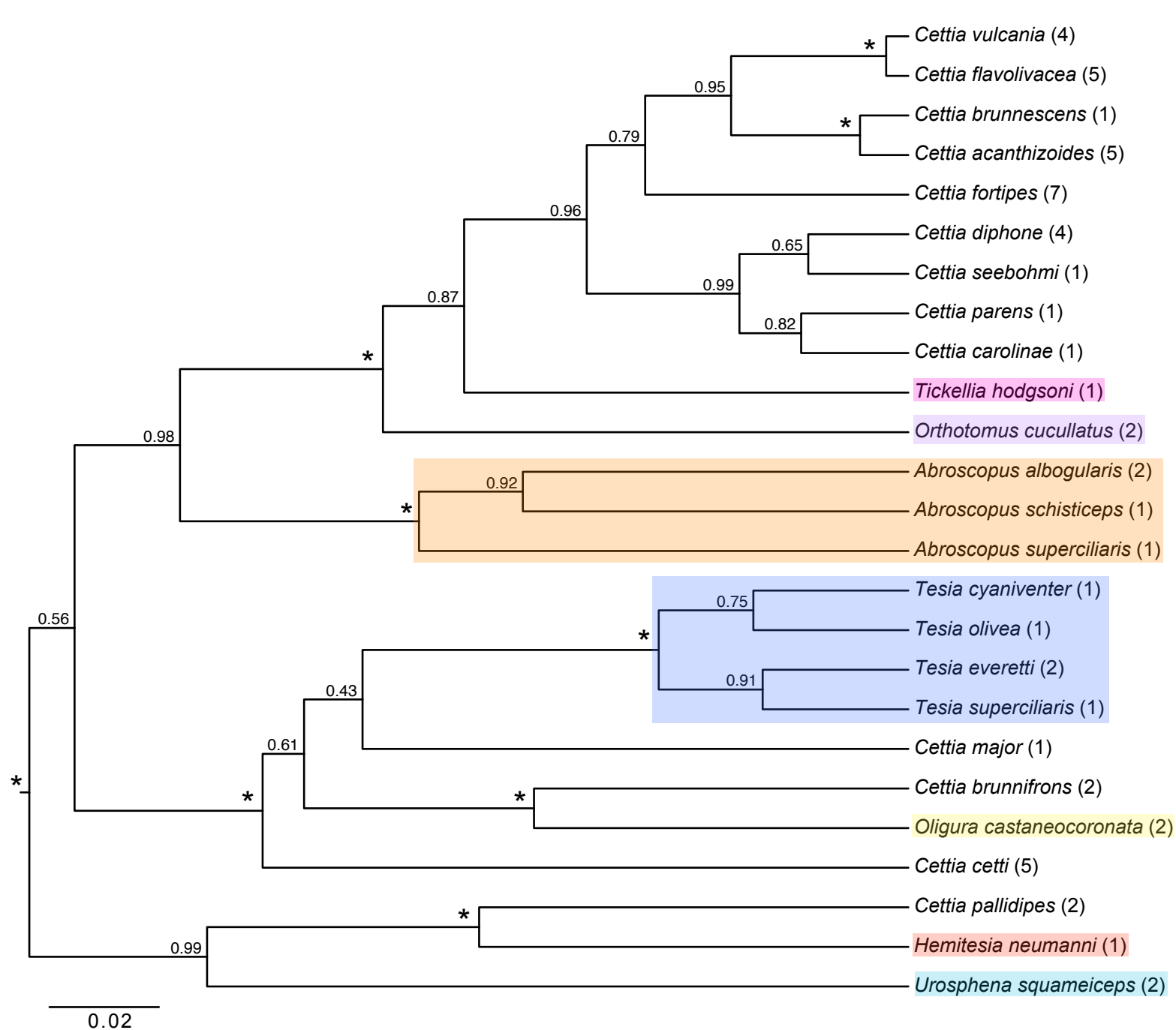

Supplement: Additional file 7 — Phylogeny of Cettiidae. Inferred by *BEAST. Only individuals for which all loci were available were included (cf. Figure 3). All loci had independent substitution models; and a relaxed clock prior was applied ("Full jModelTest relaxed"). Generic affinity according to traditional taxonomy [12] is indicated by different colour shadings. Values in parentheses after names are the number of individuals included. Posterior probabilities are indicated at the nodes; an asterisk represents posterior probability 1.00. [file 1471-2148-11-352-S7.PDF]
