# Supplementary material for: Non-monophyly and intricate morphological evolution within the avian family Cettiidae revealed by multilocus analysis of a taxonomically densely sampled dataset
Source: BMC Evol Biol. 2011 Dec 5;11:352. doi: 10.1186/1471-2148-11-352 (PMC3261208; doi:10.1186/1471-2148-11-352)
Supplement: Additional file 8 — List of samples (in alphabetical order), with GenBank accession numbers. [file 1471-2148-11-352-S8.PDF]

Additional file 8 - List of samples (in alphabetical order), with GenBank accession numbers

Species	Subspecies	Locality	Sample No./Ref.	GenBank No.			
				**Cytb**	**ODC**	**Myo**	**GAPDH**
Alauda arvensis	*arvensis*	Sweden	NRM 966614	AY228047	EF625336	AY228284	FJ357913
Abroscopus albogularis	*fulvifacies*	Sichuan, China	DZUG U1932	HQ706175	HQ706303	HQ706303	HQ706303
Abroscopus albogularis	*fulvifacies*	Taiwan	DZUG U1336	**JN808896**	**JN808995**	**JN809056**	**JN808945**
Abroscopus schisticeps	*ripponi*	Yunnan, China	DZUG U2533 / 4	**JN808897**	**JN808996**	**JN809057**	**JN808946**
Abroscopus schisticeps	*ripponi*	Yunnan, China	DZUG U2534 / 5	**JN808898**	**JN808997**	–	**JN808947**
Abroscopus schisticeps	*ripponi*	Yunnan, China	DZUG U2535 / 6	**JN808899**	**JN808998**	–	**JN808948**
Abroscopus superciliaris	*superciliaris*	Myanmar	NMNH B02119	DQ008515	EU680702	DQ008567	HQ121540
Cettia acanthizoides	*acanthizoides*	Sichuan, China	NRM 20056598 / 93_1	DQ364116	**JN809000**	**JN809058**	**JN808949**
Cettia acanthizoides	*acanthizoides*	Sichuan, China	DZUG U2337 / 93_2	–	**JN809001**	–	–
Cettia acanthizoides	*acanthizoides*	Sichuan, China	NRM 20056599 / E99	DQ364117	**JN809002**	DQ364129	DQ364124
Cettia acanthizoides	*acanthizoides*	Fujian, China	NRM 20056600 / 1	DQ364114	**JN809003**	DQ364127	DQ364122
Cettia acanthizoides	*acanthizoides*	Fujian, China	NRM 20056601 / 2	DQ364115	**JN809004**	DQ364128	DQ364123
Cettia acanthizoides	*concolor*	Taiwan	TESRI 344	DQ364118	**JN809005**	DQ364130	DQ364125

Cettia annae	monotypic	Palau	GenBank: DQ288971	DQ288971	–	–	–
Cettia brunnescens	monotypic	West Bengal, India	NRM 20056602	DQ364119	**JN808999**	DQ364131	DQ364126
Cettia brunnifrons	monotypic	Nepal	DZUG U1331	**JN808929**	**JN809040**	**JN809077**	**JN808979**
Cettia brunnifrons	monotypic	Sichuan, China	DZUG U2539	**JN808930**	**JN809041**	**JN809078**	**JN808980**
Cettia carolinae	monotypic	Tanimbar, Indonesia	RMNH 83338	**JN808922**	**JN809032**	**JN809071**	**JN808973**
Cettia cetti	*albiventris*	Punjab, India	NRM 20046810	DQ008509	**JN809030**	DQ008561	DQ364121
Cettia cetti	*albiventris*	Kazakhstan	DZUG U175	**JN808921**	**JN809031**	**JN809070**	**JN808972**
Cettia cetti	*cetti*	France	DZUG U1964 / VdT1	**JN808916**	**JN809025**	**JN809069**	**JN808968**
Cettia cetti	*cetti*	France	DZUG U1936 / VdT2	HQ121525	HQ121555	HQ121534	HQ121543
Cettia cetti	*cetti*	France	DZUG U1966 / VdT3	**JN808917**	**JN809026**	–	**JN808969**
Cettia cetti	*cetti*	France	DZUG U2041 / Ho	**JN808918**	**JN809027**	**–**	**JN808970**
Cettia cetti	*cetti*	Spain	NRM 20066034	DQ673876	**JN809028**	–	–
Cettia cetti	*orientalis*	Armenia	DZUG U1037	**HQ333039**	**HQ333087**	**HQ333072**	**HQ333101**
Cettia cetti	*orientalis*	Armenia	DZUG U1060	**JN808920**	–	–	–
Cettia cetti	*orientalis*	Armenia	DZUG U1397	**JN808919**	**JN809029**	**–**	**JN808971**
Cettia diphone	*borealis*	Russia	UWBM 75317 / 518	HQ121522	HQ121550	HQ121531	HQ121536

Cettia diphone	*borealis*	Russia	UWBM 74811 / 519	**JN808925**	**JN809036**	**JN809074**	–
Cettia diphone	*borealis*	Korea	GenB: AB159198	AB159198	–	–	–
Cettia diphone	*borealis*	Korea	GenB: AB159199	AB159199	–	–	–
Cettia diphone	*cantans*	Japan	DZUG U2540 / XJ86204	**JN808924**	**JN809034**	**JN809073**	**JN808975**
Cettia diphone	*cantans*	Japan	GenB: AB159195	AB159195	–	–	–
Cettia diphone	*cantans*	Japan	GenB: AB159194	AB159194	–	–	–
Cettia diphone	*cantans*	Miyake, Japan	DZUG U1354	**JN808923**	**JN809033**	**JN809072**	**JN808974**
Cettia diphone	*canturians*	Shaanxi, China	NRM 20046811	DQ008510	**JN809035**	DQ008562	**JN808976**
Cettia flavolivacea	*flavolivacea*	West Bengal, India	NRM 20066038	DQ673883	**JN809012**	DQ673896	**JN808956**
Cettia flavolivacea	*intricata*	Sichuan, China	NRM 20066035 / E99	DQ673880	HQ121551	DQ673893	HQ121537
Cettia flavolivacea	*intricata*	Sichuan, China	DZUG U801 / E0619	**JN808904**	–	–	–
Cettia flavolivacea	*intricata*	Sichuan, China	DZUG U1329 / E0504_1	**JN808905**	–	–	–
Cettia flavolivacea	*intricata*	Sichuan, China	DZUG U1330 / E0504_2	**JN808906**	–	–	–
Cettia flavolivacea	*intricata*	Yunnan, China	DZUG U1407 / 1407	**JN808907**	**JN809014**	**JN809063**	**JN808958**
Cettia flavolivacea	*intricata*	Yunnan, China	DZUG U1408 /	**JN808908**	**JN809015**	**JN809064**	–

			1408				
Cettia flavolivacea	*intricata*	Yunnan, China	DZUG U2541 / Y1	**JN808909**	**JN809016**	**JN809065**	**JN808959**
Cettia flavolivacea	*oblita*	Tonkin, Vietnam	NRM 20066036 / 1	DQ673881	**–**	–	–
Cettia flavolivacea	*oblita*	Tonkin, Vietnam	DZUG U2543 / 2	**JN808910**	**JN809017**	–	**JN808960**
Cettia flavolivacea	*weberi*	Chin Hills, Myanmar	NRM 20066037	DQ673882	**JN809013**	DQ673895	**JN808957**
Cettia fortipes	*davidiana*	Sichuan, China	NRM 20066040	DQ673885	**JN809009**	DQ673898	**JN808953**
Cettia fortipes	*davidiana*	Hongkong, China	DZUG U2564	**JN808902**	**JN809010**	**JN809061**	**JN808954**
Cettia fortipes	*davidiana*	Tonkin, Vietnam	DZUG U2207	**HQ333040**	**HQ333088**	**HQ333073**	**HQ333102**
Cettia fortipes	*fortipes*	West Bengal, India	NRM 20066039	DQ673884	**JN809007**	DQ673897	**JN808951**
Cettia fortipes	*fortipes*	Chin Hills, Myanmar	DZUG U2544	**JN808901**	**JN809008**	**JN809060**	**JN808952**
Cettia fortipes	*pallida*	N Pakistan	DZUG U2545	**JN808900**	**JN809006**	**JN809059**	**JN808950**
Cettia fortipes	*pallida*	Kashmir, India	GenBank: L77122	L77122	–	–	–
Cettia fortipes	*robustipes*	Taiwan	DZUG U1334	**JN808903**	**JN809011**	**JN809061**	**JN808955**
Cettia haddeni	monotypic	Bougainville Island, PNG	GenBank: DQ066451	DQ066451	–	–	–
Cettia major	*major*	Sichuan, China	DZUG U2552	**JN808931**	**JN809042**	**JN809079**	**JN808981**
Cettia pallidipes	*laurentei*	NW Thailand	DZUG U1323	**JN808914**	**JN809024**	**JN809068**	**JN808967**
Cettia pallidipes	*laurentei*	Hongkong, China	DZUG U2546	**JN808915**	–	–	–
Cettia pallidipes	*pallidipes*	Nepal	DZUG U1608	**JN808913**	**JN809023**	**JN809067**	**JN808966**

Cettia parens	monotypic	Makira, Solomon Islands	ZMUC 139455	**JN808926**	**JN809037**	**JN809075**	**JN808977**
Cettia parens	monotypic	Makira, Solomon Islands	ZMUC 139461	**JN808927**	**JN809038**	–	–
Cettia ruficapilla	*funebris*	Fiji	GenBank: DQ288968	DQ288968	–	–	–
Cettia ruficapilla	*funebris*	Fiji	GenBank: DQ288969	DQ288969	–	–	–
Cettia seebohmi	monotypic	Luzon, Philippines	DZUG U2547	**JN808928**	**JN809039**	**JN809076**	**JN808978**
Cettia vulcania	*flaviventris*	Sumatra, Indonesia	DZUG U2549 / 12	**JN808911**	**JN809019**	–	**JN808962**
Cettia vulcania	*flaviventris*	Sumatra, Indonesia	NRM 20066042 / 13	DQ673877	**JN809020**	DQ673890	**JN808963**
Cettia vulcania	*flaviventris*	Sumatra, Indonesia	DZUG U2551 / 14	**JN808912**	**JN809021**	**JN809066**	**JN808964**
Cettia vulcania	*oreophila*	Borneo, Malaysia	NRM 20066043	DQ673879	**JN809018**	DQ673892	**JN808961**
Cettia vulcania	*vulcania*	Java, Indonesia	NRM 20066041	DQ673878	**JN809022**	DQ673891	**JN808965**
Hemitesia neumanni	monotypic	Congo	NRM 570210	HQ121520	HQ121549	HQ121530	HQ121535
Mirafra javanica	*williamsoni*	Thailand	NRM 20046819	DQ008520	HQ333089	DQ008571	HQ333106
Oligura castaneocoronata	*castaneocoronata*	Nepal	DZUG U2553 / UO	**JN808932**	**JN809043**	**JN809080**	**JN808982**
Oligura castaneocoronata	*castaneocoronata*	Nepal	AMNH JGG1170	–	–	–	**JN808984**
Oligura castaneocoronata	*castaneocoronata*	Nepal	DZUG U2599 / UJ	–	**JN809045**	–	–
Oligura castaneocoronata	*castaneocoronata*	Himachal Pradesh, India	VH B0683	**JN808933**	**JN809044**	**JN809081**	**JN808983**

Orthotomus cucullatus	*coronatus*	Guangxi, China	KUNHM 96522	**JN808940**	**JN809054**	–	**JN808992**
Orthotomus cucullatus	*coronatus*	Hongkong, China	DZUG U1642	**JN808941**	–	–	–
Orthotomus cucullatus	*cucullatus*	Sumatra, Indonesia	NRM 20046815	DQ008514	–	DQ008566	–
Orthotomus cucullatus	*hedymeles*	S Sulawesi, Indonesia	DZUG U2559	HQ121523	HQ121552	HQ121532	HQ121539
Orthotomus cucullatus	*philippinus*	Luzon, Philippines	DZUG U2557	**JN808939**	**JN809053**	**JN809087**	**JN808991**
Orthotomus cucullatus	*stentor (or riedeli)*	C Sulawesi, Indonesia	DZUG U2560	**JN808943**	–	–	**JN808993**
Orthotomus sepium	*sepium*	Java, Indonesia	DZUG U803	**JN808895**	**JN808994**	**JN809055**	**JN808944**
Orthotomus sutorius	*inexpectatus*	NW Thailand	NRM 20046795	DQ008491	HQ333092	DQ008542	HQ333109
Prinia familiaris	monotypic	Java, Indonesia	NRM 20046794	DQ008490	HQ121557	DQ008541	HQ121547
Tesia cyaniventer	monotypic	South Annam, Vietnam	DZUG U1338	**JN808934**	**JN809046**	**JN809082**	**JN808985**
Tesia everetti	*everetti*	Timor, Indonesia	DZUG U2561 / 3	**JN808935**	**JN809047**	**JN809083**	**JN808986**
Tesia everetti	*everetti*	Timor, Indonesia	DZUG U2562 / 4	**JN808936**	**JN809048**	**JN809084**	**JN808987**
Tesia olivea	monotypic	Yunnan, China	NRM 20046813	DQ008512	HQ121554	DQ008564	HQ121542
Tesia superciliaris	monotypic	Java, Indonesia	DZUG U2563	**JN808937**	**JN809049**	**JN809085**	**JN808988**
Tickellia hodgsoni	monotypic	West Bengal, India	NRM 20046814	DQ008513	EU680774	DQ008565	HQ121538
Urosphena squameiceps	*squameiceps*	Japan	GenBank: AB159177	AB159177	–	–	–
Urosphena squameiceps	*squameiceps*	Japan	GenBank: AB159178	AB159178	–	–	–
Urosphena squameiceps	*squameiceps/ussuriana*	Vietnam	NRM 20026654 /	**JN808938**	**JN809050**	**JN809086**	**JN808989**

			VNM02				
Urosphena squameiceps	*squameiceps/ussuriana*	Vietnam	NRM 20046877 / VNM04	HQ121524	HQ121553	HQ121533	HQ121541
Urosphena squameiceps	*ussuriana*	Hebei, China	NRM 20046812	DQ008511	**JN809051**	DQ008563	–
Urosphena squameiceps	*ussuriana*	Korea	GenBank: AB159179	AB159179	–	–	–
Urosphena whiteheadi	monotypic	Borneo, Malaysia	NRM 569233	–	**JN809052**	–	**JN808990**

Taxonomy follows [12], except for splitting of *Cettia brunnescens* from *C. acanthizoides* [18]. AMNH = American Museum of Natural History, New York, USA; DZUG = Department of Zoology, University of Gothenburg, Göteborg, Sweden; KUNHM = University of Kansas Natural History Museum, Lawrence, Kansas, USA; NMNH = National Museum of Natural History, Smithsonian Institution, Washington, D.C., USA; NRM = Swedish Museum of Natural History, Stockholm, Sweden; RMNH = Rijksmuseum van Natuurlijke Histoire, Leiden, The Netherlands; TESRI = Taiwan Endemic Species Research Institute, Chi-chi, Taiwan; UWBM = University of Washington Burke Museum, Seattle, USA; VH = Vogelwarte Hiddensee, Zoological Institute and Museum, Ernst Moritz Arndt University of Greifswald, Greifswald, Germany; ZMUC = Zoological Museum of the University of Copenhagen, Copenhagen, Denmark. Sequences that are new to this study are in bold. In the figures, the samples are identified by locality (except where all samples are from the same locality) and/or GenBank or sample number.
